# How Does Myofascial Physical Therapy Attenuate Pain in Chronic Pelvic Pain Syndrome?

**DOI:** 10.1155/2019/6091257

**Published:** 2019-12-12

**Authors:** Keren Grinberg, Irit Weissman-Fogel, Lior Lowenstein, Liora Abramov, Michal Granot

**Affiliations:** ^1^Faculty of Social Welfare and Health Sciences, University of Haifa, Haifa, Israel; ^2^The Department of Nursing, Ruppin Academic Center, Emek Hefer, Israel; ^3^Department of Physical Therapy, Faculty of Social Welfare and Health Sciences, University of Haifa, Haifa, Israel; ^4^The Department of Obstetrics and Gynecology, Rambam Medical Center and Faculty of Medicine, Technion, Haifa, Israel; ^5^Lis Maternity Hospital, Tel Aviv Sourasky Medical Center, Sackler Faculty of Medicine, Tel Aviv University, The Sex Therapy Clinic, Tel Aviv, Israel; ^6^The Laboratory of Clinical Neurophysiology, Faculty of Medicine, Technion, Haifa, Israel

## Abstract

**Background:**

Chronic pelvic pain syndrome (CPPS) is a multifactorial disorder comprising structural and functional muscular abnormalities, a dysfunctional pain system, and psychological distress. Myofascial physical Therapy (MPT) that is targeted at improving pelvic muscle functioning is considered a first line nonpharmacological treatment for CPPS, although the precise mechanisms that lead to symptoms alleviation have not yet been elucidated.

**Purpose:**

This longitudinal study aimed to examine the local and systemic effects of MPT intervention, including biopsychophysiological processes, among CPPS patients.

**Methods:**

The study included 50 CPPS women. Morphologic assessment of the levator ani and quantitative sensory testing of the pain system were applied alongside with evaluation of pain-related psychological factors using designated questionnaires. All measures were evaluated both before and after MPT in 39 patients. The long-term effects of MPT were evaluated by clinical pain reports obtained at 3 and 9 months following MPT that were compared with a nontreated group of 11 untreated CPPS women.

**Results:**

Along with an improvement in the clinical pain intensity (*p* = 0.001) and sensitivity to experimental pain tests (*p* = 0.001) following MPT, the results also indicate that MPT has anatomical, psychological, and social therapeutic effects (*p* = 0.04; *p* = 0.001; *p* = 0.01, respectively). Furthermore, clinical pain evaluation at 3 and 9 months after MPT revealed a significant improvement in women who received treatment (*p* = 0.001).

**Conclusions:**

The findings of this pilot study suggest multisystemic (direct and indirect anatomical, neurophysiological, and psychological) effects of MPT on the multifactorial pain disorder of CPPS and therefore place MPT as a mechanism-based intervention.

## 1. Background

Chronic pelvic pain syndrome (CPPS) is defined as a multifactorial pain disorder that localizes to the anatomic pelvis, anterior abdominal wall at or below the umbilicus, the lumbosacral back or the buttocks. It is of sufficient severity to cause functional disability that may require medical care [1–6]. Structural and functional muscular abnormalities have been suggested as key features of CPPS pathogenesis, specifically hypertonicity of the pelvic floor muscles [7–10], trigger points (TrPs) in the vulvar area [11–13], and shortening of the levator muscles [14]. There are subgroups of CPPS including provoked vestibulodynia (PVD) referred to pain provoked by touch or during vaginal intercourse (dyspareunia) [15, 16] and painful bladder syndrome (PBS) characterized by pelvic pain and urinary storage symptoms (e.g., persistent urge to void, nocturia, and urinary frequency) [17, 18].

CPPS women also characterize by dysfunctional pain system as expressed by hypersensitivity of the peripheral and central pain systems, as well as dysfunctional pain modulation [3, 19–22] as well as psychological distress [23, 24], manifested as high levels of anxiety, pain catastrophizing, depression, and somatization [22, 25–33]. All these factors and the interplay between them may affect the severity of symptoms presented by chronic pain patients such as those with CPPS [34–36].

Hence, the present study is based on the biopsychosocial health model which links between biological, psychological, and social factors in understanding health and disease [37, 38]. From a biological point of view, this model refers to defects in parts and body parts and to functional impairment of body systems. In the psychological dimension, the model relates to the type of personality, attitudes and beliefs, ability to cope, and emotions such as fear, anxiety, depression, anger, and morbid behavior. In the social aspect, it relates to relationships with family members, friends, and work relations, the framework of work, medical advice, support frameworks, emotional and financial compensation, cultural factors, and socioeconomic factors. The perspective of this model serves to explore the complex mechanisms involved in chronic pain disease [39–41].

The dysfunctional pelvic floor muscle, whether originating from the lesioned muscular tissue or secondary to abnormal functioning of the pain system or psychological distress, is the target of physical therapy. This is mainly in the form of myofascial physical therapy (MPT) [42, 43]. MPT involves skillful, hands-on maneuvers directed towards relaxation, elongation, stretching, and massaging of tightened muscles, as well as the relief of myofascial tender points [44, 45]. In addition to these local effects on the pelvic floor, pain attenuation following MPT may be attributed to processes that occur at the systemic pain level, i.e., in the spinal and supraspinal structures. The latter include changes in the activity of the sympathetic nervous system and induction of pain inhibitory effects via supraspinal pathways [46, 47]. We have recently reported that MPT results in attenuation of vulvar pain and pain evoked at trigger points [48]. However, little is currently known on how MPT works and whether reductions in pain and clinical symptoms are associated with improved functioning of the pain system, as well as psychological well-being. The psychological factors, such as somatization, depression, and anxiety, were altered following pain reduction treatments and most often decreased [12, 22, 27, 36]. However, no studies have yet examined the changes of such parameters following MPT in CPPS patients.

The main goal of this prospective longitudinal preliminary study was to shed some light on the biopsychophysiological processes associated with MPT which lead to pelvic pain relief. Our investigational approach included a comprehensive evaluation of both local (morphological parameters of the levator ani pelvic floor muscle) and systemic changes (pain processing and modulation measures, as well as psychological factors) before and after MPT in CPPS patients. An additional goal was to assess the trajectory of the long-term pain relieving effects of MPT.

## 2. Methods

The data reported in this paper are part of a longitudinal study that examined the prediction and consequences of MPT in CPPS patients [48].

### 2.1. Study Participants

In this prospective longitudinal study, women diagnosed with CPPS were recruited from the Urogynecology and Pain Clinics at the Rambam Health Care Campus and the Sex Therapy Clinic at the Lis Maternity Hospital. The nontreated by MPT or any other treatment group included age-matched CPPS women (for more detailed information about the study cohort please refer Grinberg et al., 2017) [3]. Inclusion criteria for CPPS were as follows: age >18 years; for painful bladder syndrome (PBS) that is characterized by pelvic pain and urinary storage symptoms: urinary frequency ≥10 times per 24 hours, including one night-time voiding, and complaints of bladder pain ≥3 months [17, 18] for provoked vestibulodynia (PVD); and a distressing genital pain condition provoked by touch and one of the most common causes of pain during intercourse (dyspareunia) in premenopausal women [15, 16]: pain intensity during intercourse ≥4 on a 0–10 numerical pain scale (NPS) during the previous month. Patients were excluded from the study if they had a history of pelvic cancer or radiation therapy; had undergone pelvic or abdominal surgery; suffered from a urinary tract infection within the last month; or had fibromyalgia, irritable bowel syndrome, a neurological disorder, diabetes, or pregnancy. Sociodemographic and medical data including age, marital status, religion, employment status, duration of CPPS symptoms, and pharmacological treatments were recorded.

### 2.2. Experimental Procedure

The Rambam Medical Center (Haifa, Israel) Review Board, in accordance with the Helsinki Declaration and the IRB of the Haifa University, approved the study. All participants received detailed explanation and provided written informed consent before the start of any testing procedure. All subjects who agreed to participate in the study were instructed to adhere to the study protocol and did not take any medication during treatment. All women were diagnosed by a physician experienced in the field of urogynecology and dyspareunia who performed the clinical evaluation of the pelvic floor and the assessment of vulvar pain (LL and LA). Morphologic assessment of the levator ani was performed by a gynecologist specializing in ultrasonography of the pelvic floor. The psychophysical experimental procedure was conducted during the morning hours by the same investigator (KG) in the Laboratory of Clinical Neurophysiology at the Faculty of Medicine of the Technion (Haifa, Israel).

Women were initially exposed to a training session in order to familiarize them with the psychophysical tests. Thereafter, they completed the sociodemographic and medical history forms and clinical pain and urinary symptoms questionnaires, as well as the psychological questionnaires. They then underwent the psychophysical pain tests. This battery of tests was repeated again on the same women after completion of the 8 weekly session of MPT.

The study's long-term follow-up was performed after 3 months and again after 9 months (since the end of the treatment). At both time points, patients report their level of pelvic pain intensity using the 0–10 numerical pain scale (NPS) where 0 indicated “no pain” and 10 represented “the worst pain imaginable.” CPPS patients who had enrolled in the first stage of the study but decided not to have MPT served as the nontreated group. They were also asked to similarly report their 0–10 level of pelvic pain intensity at 3 and 9 months following the baseline evaluation session.

### 2.3. Clinical Evaluation of Symptom Severity

The severity of urgency symptoms among the PBS patients was assessed using the validated Hebrew version of the Urgency, Severity, and Impact life Questionnaire (USIQ) [20]. This questionnaire consists of 13 Likert scale items relating to the intensity of urgency symptoms and the severity of frequency symptoms, as well as the impact of these symptoms on daily life. The *α* Cronbach of the Hebrew version is 0.85–0.90.

The study's approach to evaluating the severity of vulvar pain symptoms and evaluation pain evoked at pelvic TrPs has been detailed in an earlier report [49].

### 2.4. Morphologic Assessment of the Levator Ani

**Figure 1:**
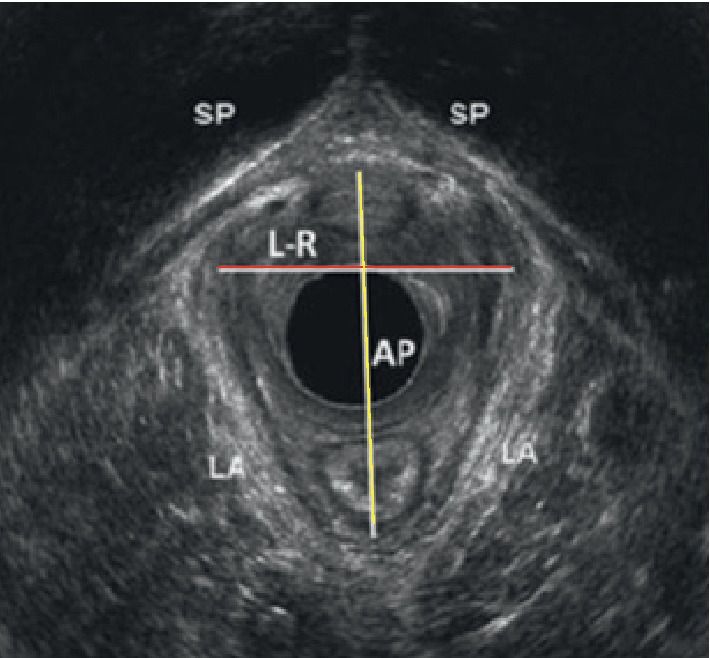
An Hiatus biometry measurement on 3D endovaginal ultrasound, intact levator ani muscle. AP, anteroposterior; L-R, left-to-right width; SP, symphysis pubis; LA, levator ani.

The length and width of the levator ani muscle was measured by using a specially designed 3D endovaginal ultrasound (BK Medical Ultrafocus machine (Peabody, MA)), device at a frequency of 4–8 MHz, as depicted in Figure 1. This ultrasound imaging was performed while the women was laying in the dorsal lithotomy position and with the hips flexed and slightly abducted, when a probe is inserted into the vagina. No preparation was required, and the patients were recommended to have a comfortable volume of urine in the bladder. This assessment was performed before and after the MPT by a gynecologist who specializes in ultrasonography evaluation. This test has been extensively described in the article of Rostamina et al. and found to be reliable [49].

### 2.5. Psychophysical Assessment of Pain

In order to examine the changes in pain sensitivity at the spinal and supraspinal level following MPT, noxious stimuli were delivered to the suprapubis area and forearm, respectively. Notably, no side effect was observed after psychophysical testing. The battery of psychophysical tests (see Grinberg et al., 2017) [3] are as follows:

#### 2.5.1. Mechanical Pain Threshold (MPT)

This was assessed at the referred area (i.e., the suprapubis area) by using von Frey hairs filaments (VFH; Stoeteling Ltd., USA) that evoke pinprick sensation, using the method of levels in an ascending order starting from the lightest VFH force of 3.7 g [50]. The lightest gram weight filament that evoked pain sensation in two out of three trials was considered to be the MPT.

#### 2.5.2. Heat Pain Threshold (HPT)

This was measured via the thermal sensory analyzer (TSA, Medoc, Ramat Yishay, Israel) with a 30 × 30 mm probe, which delivers a contact heat stimulus. Patients received three successive ramps of gradually increasing temperatures delivered to the volar forearm of their dominant hand according to the method of limits [51]. The mean of three successive responses whose variance was less than 0.5°C was calculated as the HPT.

#### 2.5.3. Magnitude Estimation of Painful Mechanical Stimulus

Three stimuli were delivered with a 225 g VFH to the suprapubis area, and women were asked to rate the level of pain intensity on a 0–100 NPS. An average of the three pain ratings was defined as the suprathreshold pain rating.

#### 2.5.4. Magnitude Estimation of Tonic Heat Pain (THP) Stimulation

A thermal stimulus at a temperature perceived to be an intensity of pain rated 50 on a 0–100 NPS (i.e., “pain 50” intensity) was applied to the volar forearm of the dominant hand for one minute. The mean ratings (NPS) of pain at 5, 25, 40, and 55 sec were defined as the suprathreshold pain.

Temporal summation of pain , a sensory phenomenon that represents central sensitization and the functioning of the facilitatory pain pathways:

- *Mechanical Temporal Summation (mTS).*Two series of 10 repetitive stimuli with a 160 g VFH were applied to the suprapubis with 2-3 sec interstimulus intervals. The differences in 0–100 NPS pain scores between the last and the first stimulus were calculated for each series and averaged across series to calculate the mTS.
- *Heat Temporal Summation (hTS).* Magnitude was calculated as the difference between the last (at 55 sec) and first 0–100 NPS pain scores (at 5 sec) that were given during the one minute of THP.

#### 2.5.5. The Conditioned Pain Modulation Paradigm (CPM)

This is an advanced psychophysical paradigm to test the efficacy of a dominant supraspinal descending endogenous analgesia mechanism, namely diffuse noxious inhibitory control (DNIC) [51, 52]. This mechanism is based on the “pain inhibits pain” phenomenon, which requires a remote noxious “conditioning stimulus” for pain attenuation of the “test stimulus.” The test stimulus was the 60 sec THP described above. The conditioning stimulus was a 90 sec immersion of the nondominant hand in a hot water bath (46.5°C) (Heto Cooling Bath, Jouan Nordic A/S, Allerod, Denmark). The test stimulus was first given alone, and pain ratings were obtained every 10 sec. After a 10 min break (to allow for nociceptor recovery to resting state), the patients were exposed to the conditioning stimulus for 30 sec after which they immediately rated the pain intensity of the hot water. Then, concomitantly with the conditioning stimulus (hot water bath), the same test stimulus was delivered again and pain ratings of the test stimulus were again recorded every 10 sec. The CPM effect was calculated as the average pain rating for the test stimulus given with the conditioning stimulus minus the average pain rating of the test stimulus given alone. A negative CPM value was considered to indicate effective endogenous pain modulation.

### 2.6. Psychological Evaluation

Participants completed a battery of psychological questionnaires including (i) the State-Trait Anxiety Inventory [53] using the validated Hebrew version [54]. The first part of the questionnaire assesses the level of state anxiety, and the second part assesses trait anxiety. Each part includes 20 statements that describe the emotional condition (reliability of 0.82–0.91). CPPS patients were asked to rate their feelings about each sentence on a 4 point Likert scale; (ii) the Hebrew version of the Brief Symptom Inventory (BSI) [55], translated by Canetty et al. [56], which assesses the level of somatization symptoms and consists of 13 self-report questions on psychological distress, (reliability of 0.78–0.91); (iii) the Hebrew version of the Pain Catastrophizing Scale (PCS) [57, 58]. This questionnaire includes 13 items representing the three components of pain catastrophizing: rumination, magnification, and helplessness. Items were rated on a Likert scale ranging from 0 (not at all) to 4 (all the time) with a total score range from 0 to 52, (reliability of 0.86); and (iv) the Beck Depression Inventory (BDI), which assesses both the cognitive and affective symptoms of depression [59]. In the BDI, a score of 0–9 is normal, 10–18 means mild depression, scores of 19–26 represent moderate depression, and scores over 26 are considered to indicate severe depression symptoms (reliability of 0.84).

### 2.7. MPT Intervention

MPT was carried out with the goal to restore pelvic floor muscle length and strength; release TrPs in the muscles and connective tissues of the pelvic floor, pelvic girdle, and abdomen, using pelvic massages (performed by a specialist woman physiotherapist) [60, 61]; and improve blood flow to the pelvic area [62]. Specifically, the MPT intervention included myofascial TrPs release and connective tissue manipulation techniques (manual stretching of the trigger point region and myofascial release). [63]. Furthermore, during treatment, the women also learned control skills and how to self-train their pelvic muscles to contract and relax [64–66]. In this study, the women were scheduled for 8 weekly 1 hour treatments with one specialist pelvic physical therapist. In addition, the patients were asked to perform a minimum of two self exercises at home (every week) in order to maximize the treatment effect and maintained a self diary. The subjective effectiveness of the MPT treatment was examined 3 and 9 months following treatment termination by asking the women to rate their responses on a 0–10 scale where 0 indicated no improvement at all and 10 represented the most effective response. As mentioned previously, the pain intensity was also examined in this period (3 and 9 months following treatment termination) using the 0–10 NPS scale.

### 2.8. Statistical Analysis

Statistical analyses were performed using SPSS version 23 (SPSS Inc., Chicago, IL). Paired *t*-tests were used to examine the differences before and after MPT regarding the anatomical muscle changes. MANOVA repeated measure tests were carried out to examine the differences between the experimental pain parameters and the psychological measures following MPT. In addition, a repeated measure ANOVA followed by preplanned contrasts were conducted to examine differences in pelvic pain scores between women with CPPS who received MPT compared with CPPS women who did not receive MPT. To compare difference in the mean difference in the pain score between the before-and-after values between groups who did and did not undergo MPT, ANOVA test was used (for example, pain at 3 and 9 months, with a variable taking into account baseline pain and a variable for the treatment group). Paired *t*-tests were carried out to examine the differences between the anatomical structure of the levator ani muscle before and after MPT treatment. All statistical tests were corrected for multiple analyses using Bonferroni correction; *p* < 0.05 was considered as significant.

## 3. Results

### 3.1. Clinical Characteristics of the Patient Population

The sample included 39 CPPS patients (21 with PBS and 18 with PVD) who underwent MPT and 11 nontreated CPPS patients. The mean age of the study group was 37.9 ± 15.4 years (range 22–67 years). The mean disease duration was 5.5 ± 9 years (range 1–30 years). As previously reported, there were no significant differences between PBS and PVD patients neither in their clinical pain ratings and consequent effects on daily functioning nor in their psychophysical and psychological parameters (*p* < 0.05) [3]. Six women with PVD also reported urine symptoms, but did not meet the criteria of PBS. The CPPS sample age ranged from 22–67 years, with an average age of 37.9 (SD = 15.4; range: 22–67), disease duration of 5.5 years (SD = 9; range: 1–30), and 53% of the participants single, 41% married, 5% divorced, and 2% widowers. More than half of them had high school education (52%), 46% had an academic degree, and 2% were college graduation. Most of the women were Jewish (93%) and 7% were Christian and Muslim.

### 3.2. Changes in Outcome Measures following MPT

In line with the reduction in the severity of clinical pain that was recently reported [48], a reduction in USIQ scores was also observed (18.1 ± 4.1 before MPT versus 13.6 ± 3.4 after MPT; *p* < 0.001) together with their impact on daily functions (23.1 ± 7.0 before MPT versus 18.5 ± 7.1 after MPT; *p* < 0.01).

A significant correlation was found between the change in the pain test (CPM test) and the change in the muscle length (*r* = 0.661, *p*=0.037) and width (*r* = 0.671, *p*=0.034).

### 3.3. Changes in Psychophysical Parameters

MPT significantly impacted the experimental pain parameters as shown from analyzing the changes before and after treatment. Specifically significant reductions in pain sensitivity were observed (*F*(10, 27) = 5.26, *p* < 0.001) for the following measures: pain thresholds, suprathreshold rating of heat and mechanical pain stimuli, and CPM magnitude (Table 1).

### 3.4. Changes in Psychological Parameters

MPT significantly improved all pain-related psychological parameters before and after MPT (*F*(5,32) = 5.72, *p* < 0.001), with improvements in state and trait anxiety levels, pain catastrophizing, somatization, and depression (Table 2).

### 3.5. Changes in Anatomical Measures

Due to technical problems, data were obtained from only 11 women who were available for the ultrasonographic analysis. An increase in the width of the levator ani (*t* = 2.28, *p*=0.04) was observed following MPT. However, there were no significant changes in muscle length (Table 3). No significant correlations were found between the change in the length or width of the levator ani and the changes in the clinical pain ratings before and after treatment (*p* < 0.05).

### 3.6. The Long-Term Effects of MPT on Clinical Pain

**Figure 2:**
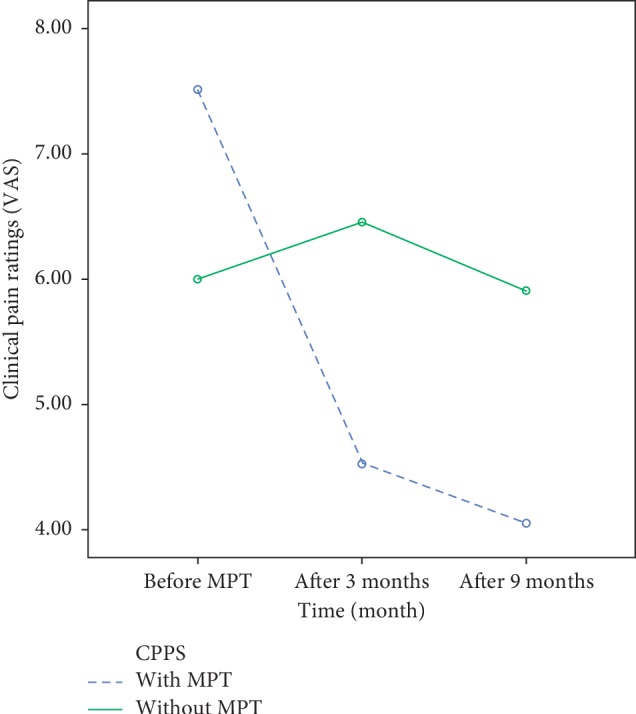
Clinical pain scores (0–10 NPS) of women with CPPS at baseline and following MPT compared with CPPS women who did not undergo MPT; ^*∗∗*^*p* < 0.01, ^*∗∗∗*^*p* < 0.001. There was a significant improvement in clinical pain ratings among women receiving MPT as assessed before the treatment (*t* = 3.18, *p*=0.003) and at 3 (*t* = 3.97, *p*=0.000) and 9 months (*t* = 3.58 *p*=0.000) compared with the nontreated group. Baseline differences in clinical pain were controlled.

Repeated measure ANOVA showed significant improvement in self reports of pelvic pain intensity 3 and 9 months following up MPT as compared with baseline pain intensity (*F*(1, 47) = 7.004, *p*=0.001), whereas, no significant change in pelvic pain was observed in the group that did not undergo MPT, and patients who completed the treatment demonstrated significant improvement and stable pain alleviations as assessed at 3 and 9 months compared with the reports of pelvic pain obtained at baseline. (Table 4 and Figure 2).

## 4. Discussion

PVD and PBS represent subgroups of CPPS. They have shared characteristics including muscular abnormalities, a dysfunctional pain system, and psychological distress [3], suggesting a common mechanism-based treatment strategy. Thus, this preliminary study was aimed at investigating how MPT, a pelvic floor-focused treatment, is able to affect biopsychological measures and alleviate symptom severity in women with CPPS from PVD or PBS. The results indicate that MPT has anatomical, neurophysiological, and psychological therapeutic effects alongside long-lasting pelvic pain alleviation. Specifically, MPT works on relieving hypertonicity, reduces the sensitivity to experimental pain, improves the functionality of the endogenous inhibitory system, and decreases psychological distress (i.e., state and trait anxiety levels, pain catastrophizing, somatization, and depressive symptoms). These systemic effects position MPT as a multisystemic therapeutic intervention for patients with CPPS.

Evidence suggests that the MPT approach improves blood flow to the pelvic region and involves the releasing of TrPs [45, 67]. MPT has also been shown to normalize muscle tone, flex the connective and soft tissue around the joints of the pelvic floor, and strengthen the pelvic girdle muscles [45, 68–75]. The fact that women in the current study who were not treated by MPT showed no significant reduction in their clinical pain intensity after 3 and 9 months reinforces the assumption that the short- and long-term effects of MPT cannot be attributed solely to a placebo effect [45, 76]. The combined positive effects on anatomical, neurophysiological, and psychological processes associated with MPT are revealed for the first time in this study.

The anatomical effect of MPT on the width of the levator ani muscle supports the improvements seen in the clinical picture. The levator ani plays a role in supporting the pelvic organs and the functional mechanism of the sphincters. Physical therapy to the pelvic region was focused on imparting patient control of the muscle relaxation and contraction processes, which may contribute to improving muscle relaxation and consequently to changing the muscle width. The literature supports such a causative linkage, as studies have reported a lengthening of the levator ani muscle following MPT in CPPS patients that was correlated with the degree of improvement in clinical pain ratings [45, 77, 78]. The current study did not compare the muscle length and width between women with CPPS and healthy women, which eliminates the possibility to derive any conclusions about a possible anatomical impairment in the muscle prior to the intervention. Yet, it has been suggested that an anatomical defect is one of the mechanisms underlying CPPS that may arise due to stress situations, past events, or perhaps originate from an organic, anatomical, or morphological impairment [4, 32]. An indirect indicator of the anatomical defect might be represented by increased pain ratings in response to contact stimuli at the TrPs, as previously reported in other studies [20, 21], as well as on this cohort [48]. Our findings of reduced pain hypersensitivity following MPT [48], accompanied by the morphological enlargement of the levator ani, may suggest that this anatomical change also contributes to the reduced sensitivity of TrPs.

MPT also affected the systemic pain sensitivity to experimental pain measures. Specifically, we observed an increase in pain thresholds and a decrease in pain ratings in response to noxious stimulation in referred and remote body areas. Two possible central mechanisms may be involved in this treatment-induced plasticity. Firstly we suggest that MPT decreases the level of central sensitization, where sensitization of neurons at the spinal and supraspinal levels contributes to the development and maintenance of referred and remote pain, respectively [79, 80]. It is therefore a fair assumption that in this study, MPT diminished the degree of hypersensitivity at the local level (the pelvic soft tissue), as expressed by a reduction in evoked pain (e.g., TrPs), and thus reduced nociceptive input to the central pain system. The diminished barrage of nociceptive information may have consequently resulted in decreased sensitization at the spinal and supraspinal levels as manifested by the reduced pain sensitivity at referred and remote body areas [20, 81–83].

The second suggested central mechanism is based on the improved endogenous inhibitory capacity following MPT, as demonstrated by an increased CPM response. CPM is the experimental paradigm to test diffuse noxious inhibitory control (DNIC), a descending system that induces top-down modulation on the nociceptive neurons at the spinal level, resulting in a systemic inhibitory effect and pain alleviation. Research has indicated a dysfunctional DNIC system in CPPS [3, 21, 83, 84] and other idiopathic pain conditions [85, 86]. Notably, interventions aimed at pain alleviation (i.e., surgical and pharmacological) have the capability to restore the functioning of the endogenous inhibitory system [85]. For example, Kosek and Ordeberg [87] identified a restoration of the DNIC system in osteoarthritis patients who underwent hip or knee surgery and reported significant pain relief. These results suggest that the dysfunctional pain inhibition is maintained over time by ongoing pain and when the pain is extinguished, and DNIC function may be restored resulting in an attenuation of pain sensitivity. We therefore suggest that following MPT treatment, improved CPM functionality was observed in our patients, probably due to a reduction in the ongoing pain, and such enhanced inhibitory activity contributed to the reduction in the clinical pain.

MPT was also associated with psychological benefits in our patient cohort, as expressed by a reduction in psychological distress and a decrease in the level of state and trait anxiety, as well as decreased ratings of depression, catastrophizing, and somatization after treatment. Previous studies indicate that a reduction in psychological distress can alleviate pain. For example, a decrease in fear, anxiety, and catastrophic thoughts is able to reduce negative feelings and somatic complaints and may affect the response to experimental and clinical pain [36, 58, 88–90]. Furthermore, high levels of catastrophizing have been attributed to clinical pain intensity and experimental pain sensitivity [34, 55, 91, 92]. The literature thus supports the present study's findings of improved pain-related personality factors in parallel with reduced clinical and experimental pain parameters. Conversely, it is also possible that the chain of events is in the opposite direction and that the reduction in clinical pain improved the psychological distress.

It is important to note an additional perspective to interpreting the observed results of MPT in CPPS patients, namely, the patient-physiotherapist relationship. This may hold therapeutic effects that are above and beyond the direct local influence on the muscular system [45, 69]. Such relationships may involve instilling a sense of control, security, and trust, as well as self-efficacy [93], which are invaluable in improving clinical outcomes of chronic pain disorders. Furthermore, frustration resulting from the failure of previous treatments may have led women to develop negative thoughts in relation to their disease, which also manifested as catastrophic thinking when relating to their pain [94]. An appropriate treatment is therefore likely to reduce such catastrophizing and indeed following MPT, improvements were found in the current study on this psychological factor. Lowered catastrophizing thinking may be also achieved due to the improvement of the patients' ability to voluntarily contract and relax the pelvic floor muscles and thus control urine functioning. This leads to the adaption of more efficient coping strategies as well as a better sense of control. With regard to the biopsychosocial model, it is therefore likely that MPT also has cognitive-psychological effects that may improve CPPS symptoms beyond its physical influence.

Several limitations that may influence the significance of the findings should be addressed. First, the relatively small sample size limits the generalization of our findings given the variability in psychophysical and pain-related personality measures. The small sample size was due to the restrictive inclusion criteria which reduced the number of CPPS patients that could be enrolled. Second, due to the fact that the 11 women who did not undergo MPT were not randomly selected and that their assignment to this group was based solely on their reports of clinical CPPS symptoms, this nontreated group is not a “true” control group. In order to present an understanding of the mechanistic processes, a real randomized control group should be obtained and tested with both psychophysical and psychological measures. Therefore, the significance of the findings should be carefully interpreted relating to this pilot study. It cannot be ignored that the ability to make decisive conclusions from the findings of this pilot study is limited.

## 5. Conclusions

This study sheds light on the indirect neurophysiological and psychological effects of MPT that occurred together with pain alleviation and improvement in functioning. The peripherals and systemic effects of MPT position it as a multisystemic therapeutic intervention for patients with CPPS. This suggestion is in line with the notion that CPPS is a multifactorial and complex pain disorder comprised of multiple biopsychosocial components. Therefore, an intervention such as MPT that has multisystemic effects can be recommended as a mechanism-based intervention for CPPS patients. We suggest that future randomized control studies conducted on larger cohorts of patients may allow the reliability of our results to be addressed.

## Figures and Tables

**Table 1:** Differences in experimental pain parameters before and after MPT.

	Pre-MPT	Post-MPT	Mean difference	*F*	*p*
MPT (gr.)	5.6 ± 0.5	5.7 ± 0.4	0.1	1.90	0.17
1^st^ supra-m (NPS)	29.7 ± 20.5	20.7 ± 15.6	−9.0	12.50	0.001
10^th^ supra-m (NPS)	41.8 ± 25.5	31.3 ± 21.3	−10.5	15.20	0.000
mTS (NPS)	10.3 ± 15.2	12.2 ± 20.3	1.9	0.24	0.62
HPT (°C)	41.1 ± 2.8	41.9 ± 2.7	0.8	4.81	0.035
Pain 50 (°C)	42.7 ± 2.3	43.2 ± 2.2	0.5	10.60	0.002
Contact THP (NPS)	46.1 ± 13.3	45.6 ± 14.2	−0.5	0.06	0.05
Immersion THP (NPS)	67.0 ± 37.9	72.8 ± 30.7	5.8	1.50	0.12
CPM (NPS)	−0.2 ± 13.9	13.8 ± 12.2	14	34.90	0.000

Data are shown as mean ± standard deviation. MPT, mechanical pain threshold; 1^st^ supra-m, the mean pain rating of the first mechanical stimuli; 10^th^ supra-m, the mean pain rating of the tenth mechanical stimuli; HPT, heat pain threshold; NPS, 0–100 numerical pain scale; mTS, mechanical temporal summation; hTS, heat temporal summation; Pain 50, the temperature that induces a pain of 50/100 on the NPS; Contact THP, the mean pain rating from the tonic heat stimulus of the thermal sensory probe; Immersion THP, the mean pain rating from the tonic heat stimulus of the hot-water bath; CPM, conditioned pain modulation.

**Table 2:** Differences in psychological factors before and after MPT.

	Pre-MPT	Post-MPT	Mean difference	*F*	*p*
Anxiety state	49.4 ± 7.0	46.6 ± 4.9	−2.8	4.42	0.043
Anxiety trait	47.7 ± 4.8	46.0 ± 5.0	−1.7	4.62	0.038
PCS	26.2 ± 13.0	21.2 ± 12.2	−5.0	7.32	0.001
BSI	13.4 ± 8.7	10.1 ± 6.5	−3.3	15.77	0.000
BDI	12.0 ± 6.8	8.0 ± 6.3	−4.0	17.72	0.000

Data are shown as mean ± standard deviation. Anxiety state and anxiety trait, from the State-Trait Anxiety Inventory; PCS, pain catastrophizing scale; BSI, brief symptom inventory; BDI, beck depression index.

**Table 3:** Anatomical structural differences before and after MPT.

	Pre-MPT (*N* = 11)	Post-MPT (*N* = 11)	Mean difference	*T*	*p*
Levator ani length (cm)	5.7 ± 0.7	5.2 ± 0.8	−0.5	1.77	0.11
Levator ani width (cm)	4.3 ± 0.7	4.8 ± 0.9	0.5	2.28	0.04

Data are shown as mean ± standard deviation.

**Table 4:** Differences in pain scores (after 3 and 9 months) of women with CPPS who received MPT compared with women with CPPS who did not receive MPT.

	CPPS with MPT (*N* = 39)	CPPS with no MPT (*N* = 11)	Mean difference	*F*	*p*
NPS at baseline	7.6 ± 1.4	6 ± 1.2	−1.6	3.25	0.31
NPS at 3 months	4.4 ± 2.3	6.5 ± 1.5	2.1	2.91	0.005
NPS at 9 months	4.1 ± 1.5	5.9 ± 1.2	1.8	3.70	0.01

Data are shown as mean ± standard deviation. NPS at 3 months, clinical pain ratings after 3 months on a 0–10 numerical pain scale; NPS at 9 months, clinical pain ratings after 9 months on a 0–10 numerical pain scale. There was a significant improvement in clinical pain scores among women receiving MPT compared to women with CPPS who did not undergo any treatment, as assessed at 3 and 9 months.

## Data Availability

The datasets generated and analyzed during the current study are available from the corresponding author on reasonable request.

## Abbreviation

CPPS: Chronic pelvic pain syndrome
MPT: Myofascial physical therapy
TrPs: Trigger points
PVD: Provoked vestibulodynia
PBS: Painful bladder syndrome
NPS: Numerical pain scale
USIQ: Urgency, severity, and impact life questionnaire
MPT: Mechanical pain threshold
VFH: Von Frey hairs
HPT: Heat pain threshold
TSA: Thermal sensory analyzer
THP: Tonic heat pain
mTS: Mechanical temporal summation
hTS: Heat temporal summation
CPM: Conditioned pain modulation paradigm
DNIC: Diffuse noxious inhibitory control
BSI: Brief symptom inventory
PCS: Pain catastrophizing scale
BDI: Beck depression inventory.

## Additional Points

Myofascial manual therapy induces anatomical, neurophysiological, and psychological effects in chronic pelvic pain patients.
